# Expanding the PHES-ODM: A Comprehensive, Open-Source Data Model for the Future of Wastewater-Based Epidemiology

**DOI:** 10.3390/microorganisms14061267

**Published:** 2026-06-04

**Authors:** Mathew Thomson, Jean-David Therrien, Nikho Hizon, Janet Ting-mei Lin, Martin Wellman, Eugen-Sorin Sion, Carol Bennett, Peter A. Vanrolleghem, Douglas Manuel

**Affiliations:** 1 Ottawa Hospital Research Institute, University of Ottawa, Ottawa, ON K1Y 4E9, Canada; matthomson@ohri.ca (M.T.);; 2 modelEAU, Université Laval, Québec City, QC G1V 0A6, Canada; 3 Faculty of Bioscience Engineering, Ghent University, 9000 Ghent, Belgium; 4 Public Health Agency of Canada National Microbiology Laboratory, Winnipeg, MB R3E 3R2, Canada; 5 Joint Research Centre, European Commission, 21027 Ispra, Italy

**Keywords:** wastewater surveillance, wastewater epidemiology, environmental surveillance, data standardization, relational data modeling, database schema, public health data, public health

## Abstract

Wastewater surveillance (WWS) has quickly emerged as an invaluable tool for public health surveillance, particularly in the wake of the COVID-19 pandemic. Its long-term utility is constrained, however, by fragmented data systems, inconsistent metadata practices, and poor interoperability. The Public Health and Environmental Surveillance Open Data Model (PHES-ODM) was developed as an open, collaborative framework to standardize WWS data and support transparent, ethical data use aligned with FAIR principles in response to these challenges. Building on the success and global adoption of earlier versions, this paper introduces version 3 of the model, expanding to address persistent barriers to interoperability and data utility. Key enhancements include improved metadata capture, support for complex relational linkages across sites, samples, measures, and populations, and new tables for public health actions, external data linkages, and analytical workflows. Tools for mapping across existing standards and supporting long and wide data formats are also introduced. Balancing robustness with usability, PHES-ODM v3 provides a scalable, modular infrastructure adaptable to diverse WWS programmes. The model offers comprehensive solutions for improving data quality, accessibility, and integration, supporting more effective public health decision-making in an increasingly complex global surveillance landscape.

## 1. Introduction

Historically used to track fecal-transmitted and water-borne pathogens, wastewater surveillance (WWS) of public health threats has become increasingly discussed since the emergence of SARS-CoV-2 and the COVID-19 pandemic [1,2]. Since then, almost 300 universities, with over 4500 sites in over 70 countries, implemented WWS programmes [3,4]. The World Health Organization, among other experts, have lauded the rapid development and adoption of WWS as a valuable addition to the existing pathogen surveillance programmes and celebrated its utility as a valuable tool for promoting and protecting human health [5,6]. As part of the boom in WWS, the Rockefeller foundation convened the Wastewater Action Group [7], and the Bill and Melinda Gates Foundation made WWS part of its Enterics, Diagnostics, Genomics & Epidemiology (EDGE) programme [8]. Voluntary and global networks-of-networks, such as GLOWACON [9], have also been assembled, with an aim to establish global networks, and networks-of-networks, to support WWS. These networks have succeeded in convening and supporting the community of practice and have fostered knowledge sharing and the deployment of innovations for informed public health decision-making [9].

The rise in WWS, however, is not without challenges [7,10]. In particular, data from WWS is not made widely accessible, and this restricted access to data hampers large-scale coordination and meta-analytical research, ultimately obstructing the integration of wastewater insights into actionable public health policies. Challenges have been persistent since the inception of many programmes [3,4]. As such, addressing barriers and improving support for open data sharing is a priority for global networks. Networks have begun to collaboratively generate and release guidance documents on how to communicate wastewater-related public health findings, as well as best practices on how to perform the analyses required. Networks are also discussing standardization, best practices in statistical modelling, and feasibility studies on open-sharing platforms [7,8,9]. For new surveillance systems like WWS, the absence of (or unwillingness to adopt) existing data standards, insufficient system literacy, lack of guidance on the interpretation of data, insufficient data infrastructure, poorly understood or explained laboratory infrastructure, and the diversity of fields involved in WWS in particular, all pose important challenges to the longevity and utility of these programmes, as well as to data sharing and coordination [11]. Furthermore, issues around consistent data reporting, the lack of interoperable data formatting, inconsistent metadata collection, and reticence toward data sharing all need to be addressed if WWS is to advance and become a permanent fixture of the public health landscape [11,12].

The Public Health and Environmental Surveillance Open Data Model (PHES-ODM) was developed during the inception of WWS for SARS-CoV-2 in Ottawa, Canada, to standardize data reporting and storage [13,14]. By structuring real-world entities and recording critical metadata—such as collection methods and analytic protocols—the model provided valuable context as diverse labs began reporting to the Ontario provincial government. From the first prototypes, this metadata was instrumental in distinguishing true infection trends from site-specific technical variations. Originally developed in collaboration with the Delatolla lab (Ottawa) and the CentrEAU research cluster (the province of Québec), the PHES-ODM filled a void where no national or international standards have yet to exist. The remarkable success and impact of open data sharing from COVID-19 clinical surveillance systems [15] and growing interest in PHES-ODM led to the adoption of a fully open and collaborative platform. Major contributing organizations included the European Commission Joint Research Centre, the United States Centers for Disease Control and Prevention (USCDC)’s National Wastewater Surveillance System (NWSS) [16], and the Public Health Agency of Canada [17], along with Canadian provincial WWS programmes in Ontario and Québec. The model was also adopted by organizations in the private sector, notably the CETo epidemiological software programme [18] and AdvanSentinel’s programme [19]. Collaboration and participation continued to expand with regular working group meetings, and membership from all world regions except Africa.

The PHES-ODM was designed as an open, collaborative data standard to provide a common language when discussing WWS globally. It emerged to fill the need for a standard or openly available structure for recording and storing WWS data. This was an issue to be solved because transparency in maintaining data is foundational to its ethical handling [20], and transparency and metadata are crucial to principles of data justice. According to D’Ignazio and Klein [21], “consider context” is the sixth principle of data feminism; without context—provided through metadata—data remains prone to misinterpretation. When this data is about humans and human health, misinterpretation can cause immense harm. Data is also only useful if it is understandable and analyzable—without access and context, there is no possibility to repurpose the data. Governments also risk squandering public funds and eroding public trust if collected data cannot be used. To better define guidelines to assess data transparency and usability, the FAIR Data Principles assert that responsible and ethically managed data should be Findable, Accessible, Interoperable, and Reusable [12]. In line with these principles, we developed an open data dictionary to support metadata management. Data dictionaries, resources that support data curation by providing standardized definitions of terms for use in metadata, are foundational to transparent data collection and management. They also facilitate ethical handling of sensitive data, and support transparency in research and data inquiry through the entire lifecycle of the data [20]. These kinds of good data management and stewardship protocols are prerequisites to advancement and innovation in any field, but particularly in developing fields such as WWS and wastewater-based epidemiology (WBE) where sharing and collaboration between areas of practice and institutions are essential.

In its approach to WWS data, the PHES-ODM emulates approaches from similar systems within public health, like Logical Observation Identifiers Names and Codes (LOINC), a universal standard and database for medical laboratory measures [22]. In this way, version 1 of the PHES-ODM enabled users to record the basic WWS data and metadata in a standardized way, facilitating exchange and aggregation to improve coordinated surveillance efforts [13]. Beyond technical utility, the model was meant to improve public health outcomes by enhancing environmental surveillance and epidemiology through interoperable, transparent, and efficient data collection and use. By using a relational database structure, the model links data across the entire analytic lifecycle. With unique identifiers for sites, samples, measures, and other attributes, the relational structure allows for each piece of the WWS process to be recorded and accounted for, while avoiding issues like data duplication, inconsistency, and challenges with insertion and deletion [14,23]. Version 1 focused on recording sample information and measures, site information and measures, and clinical surveillance information on COVID-19 infections. With version 2 of the PHES-ODM, the model collapsed site and sample measures into a singular measures table, while expanding additional provisions for methods, protocols, and sample provenance, among other fields [14]. While the original version 1 [13] could still be effective for small-scale SARS-CoV-2 surveillance programmes, version 2 went much deeper in response to growing demands for additional metadata fields. It provided templates for reporting variants and mutations of SARS-CoV-2, and the tracking and reporting of additional pathogens. The structure of version 2 built on the original relational format and expanded linkages. By integrating this relational approach with entity-relationship modelling frameworks [24], the PHES-ODM provides a robust and highly customizable database structure that ensures data provenance is explicit in the metadata.

While the adoption and use of both version 1 and 2 of the model have been very successful, global progress on WWS programmes is now at risk. As political priorities shift toward managing polycrises [25] and an endemic approach to SARS-CoV-2, there is a danger of losing the momentum gained in global wastewater surveillance [6]. A concerted effort to continue to support and develop WWS programmes and initiatives is required to maintain this system for the next pandemic. Initiatives to assess programmes are forthcoming [26], but data models need to be responsive to additional context and expanding programmes. After the release and wide global adoption of version 2 of the PHES-ODM [14], it is now the official model used by the Public Health Agency of Canada for their WWS programme [17] and has been adopted and modified by the European Union Sewage Sentinel System for SARS-CoV-2 [27], among other programmes and initiatives. WWS data is still, however, heavily siloed and in a crisis of a lack of interoperability and sharing.

Building on the foundation of version 2, this paper introduces version 3 of the PHES-ODM. The revisions and expansions in this latest iteration were developed in direct response to consultations with current users and data dictionary developers. We explore the model’s evolution, focusing on its ability to balance robust functionality with ease of use while supporting interoperability within complex epidemiological environments. Ultimately, version 3 offers structural solutions to common data challenges in WWS; this paper details the model’s architecture, target audience, implementation, and overall utility for global WWS programmes and role as a solution to data challenges in the larger field of WWS.

## 2. Materials and Methods

### 2.1. Overview of the PHES-ODM: The Structure of the Model

The PHES-ODM organizes wastewater surveillance (WWS) data around three core entities that we recommend for most purposes: sites, samples, and measures. A site is a location where wastewater is collected or where measurements are taken—for example, a wastewater treatment plant, a manhole, or a building. A sample is a discrete portion of water or wastewater collected from a site at a defined time or interval. A measure is a single observation: a PCR result, a flow rate, a temperature reading, or any other recorded value. Together, the three entities describe where the data come from, what was sampled, and what was measured.

**Figure 1:**
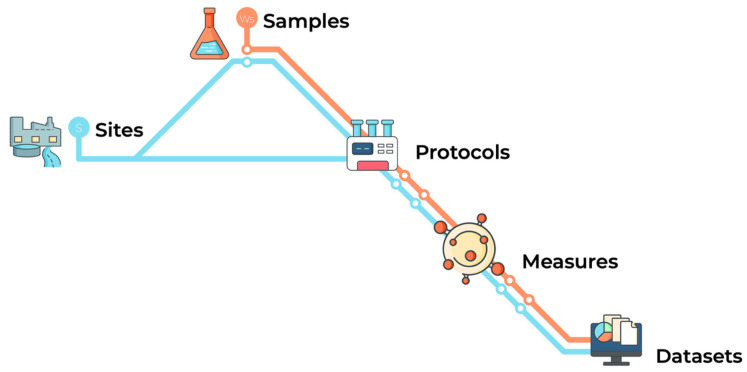
Map of the flow of wastewater surveillance (WWS) data from a site into a database. This stylized diagram shows how site information and sample information complement one another, and may also follow similar paths, but are ultimately distinct and important to WWS use and application.

Each entity is represented by a table, with attributes recorded as columns. Table 1 lists the variables most users will populate; Figure 1 illustrates how the three tables link. Many additional optional variables are available in the full model for programmes that record richer metadata. The PHES-ODM working group of international users developed a minimum set of requirements for a valid dataset, described in Section 2.2, and some of the full set of structural extensions—polygons, populations, public health actions, calculations, protocols, and others—in Section 3.4.

The relationships between these tables follow a simple hierarchy. One site can have many samples, and one sample can yield many measures—both are one-to-many relationships. An entity’s identifier is mandatory in its home table and is repeated in the related table to link rows. There is one useful exception: a measure does not always need a sample. A continuous flow measurement at a treatment plant, for instance, links directly to the site without an intervening sample.

Beyond the core three, the PHES-ODM allows for the recording of more expansive geographic and population data—distinguishing it from most other WWS data standards. Polygon records describe defined areas such as municipal boundaries, treatment-plant catchments, or health-administrative regions, encoded as closed coordinate sequences for interoperability with Geographic Information Systems [28]. Population records carry public-health outcomes such as case counts or hospitalization rates within a defined area. Together, these extensions support data storage across multiple scales:

- Polygons: Defined areas such as catchments and administrative regions;
- Sites: Technical data such as flow rate at a treatment plant;
- Samples: A discrete portion of water collected at a point or over a period;
- Measures: Any observation from a site or sample;
- Populations: Public health outcomes for a defined area.

A more detailed account of the basic structure in version 2 of the PHES-ODM and the rationale for this structure is described separately [14]. To review the general principles, however, we need to acknowledge that wastewater and environmental surveillance data are complex and that many factors, originating from vastly different disciplines, must be considered when interpreting them. Information about the geographic area, its population and composition; environmental and industrial factors in and around the sampling site; sample composition and treatment details; information on the methodology and analytical assays employed; as well as data on measurement and sample quality, are all crucial to interpreting WWS data.

As shown in Figure 1, the flow of data in the PHES-ODM goes from the sampling site through sample collection to measurement. Site information, such as geographic location and wastewater flow rate, is crucial for contextualizing WWS measurements. Sample information, such as the type of material (primary sludge, raw wastewater), is also essential when comparing measurements across programmes. Data can flow from the site through samples and protocols—a sample is taken from a site, analyzed, and a measurement is recorded—or may bypass samples entirely when a measurement is taken directly at the site (e.g., flow rate or weather conditions).

**Figure 2:**
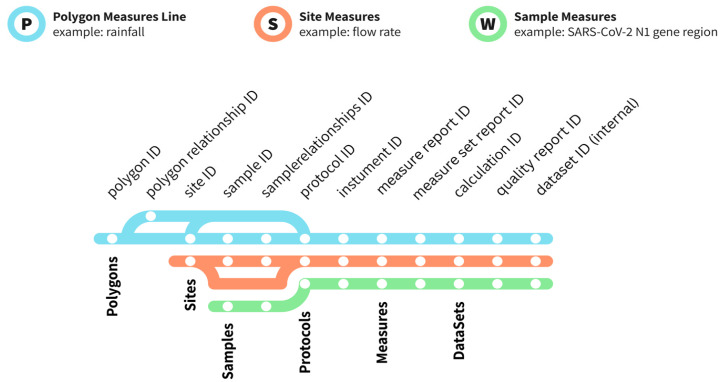
A subway-map-style diagram illustrating the flow of information in the PHES-ODM for different levels of measurement. The diagram shows how information at the levels of samples, sites, and polygons, may overlap in areas and complement each other in WWS databases. The diagram includes examples, as well as some of the IDs or keys used to link together these types of entities in practice in the PHES-ODM.

Figure 2 shows an expanded version of Figure 1, which includes a data stream for polygons and some of the identifiers used to link different parts of the model.

**Figure 3:**
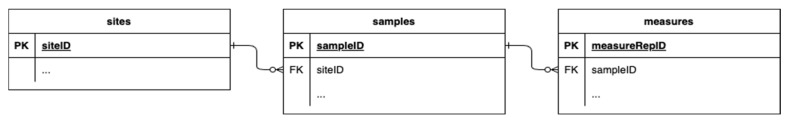
Example entity relationship diagram in wastewater surveillance demonstrating structure, relationship, and the use of keys and IDs. The figure shows how “siteID” operates as a primary key for the “sites” table, but as a foreign key to the “samples” table to facilitate linkages between sites and samples. Similarly, “sampleID” operates as a primary key for the “samples” table, but as a foreign key to the “measures” table to facilitate linkages between samples and measures.

The PHES-ODM is implemented as a relational database—a structured system in which data are organized into linked tables. The relational structure is vital for WWS, where data are inherently complex and interrelated. By separately tracking site, sample, population, and geographic information, the model provides a comprehensive overview of the surveillance landscape while eliminating the onerous data entry required for repeated items. In this framework, real-world concepts are represented as “entities” (e.g., a sample or a site), while their specific characteristics are recorded as “attributes” (e.g., collection date or building type). In practice, each entity is represented by a table, where attributes serve as column headers. The relationships between these tables are managed through unique identifiers, or keys [23,24]. For example, the “sites” table records all information about sampling sites, with “siteID” (a Primary Key (PK)). When a sample is collected at a site, “siteID” appears in the “samples” table to link location data to sample records without duplicating site information (here as a Foreign Key (FK)). This pattern repeats: “sampleID” links to the “measures” table, chaining sites to samples to measures. For an illustration of this structure, see Figure 3. The cardinalities of these relationships are often one-to-many, with one identifier for a single record in samples, sites, etc., linking to many records in other tables. For example, one site links to many samples, and one sample links to many measures. Identifiers in their home tables are always mandatory, but in other tables are often just recommended. For example, a measure of flow or water quality at a wastewater treatment plant will be linked to a site but may not be linked to a sample if this measure comes from a sensor network at the plant.

**Figure 4:**
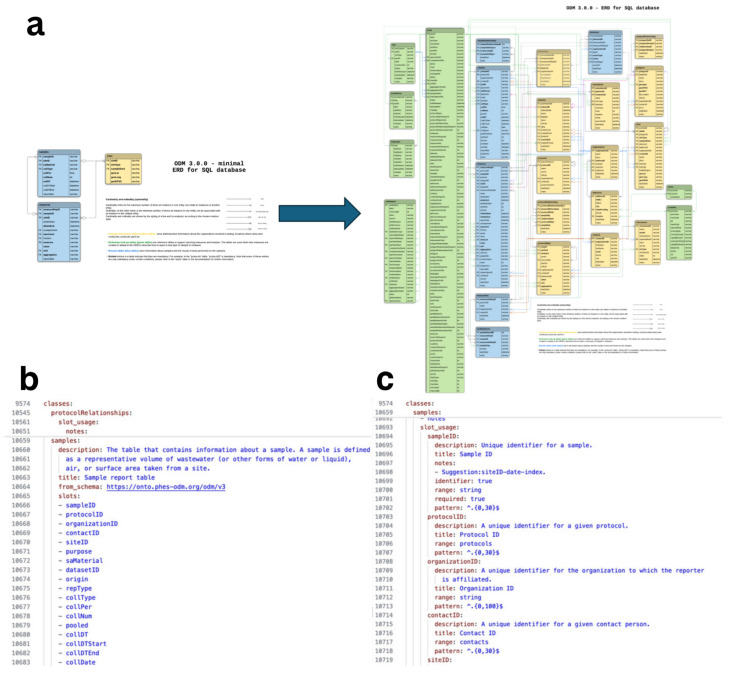
(**a**) Comparison of entity relationship diagrams between the minimal and full versions of the PHES-ODM. The minimal version (**left**) comprises only 3 essential tables. Namely, samples, measures, and sites. The relationships in this format are also much fewer. The full version of the PHES-ODM (**right**) contains all possible tables, including reference tables. The relationships are far more numerous. (**b**) The samples table in the PHES-ODM and its columns, represented as slots in LinkML. The LinkML schema for the PHES-ODM flattens tables into lists of headers (called slots), with a description, title and source for each table. (**c**) Descriptions, rules, and structure of headers (slots) in samples table of the PHES-ODM in LinkML. The LinkML schema also lists, for each header (slot), a description, title, and notes. You can also specify rules for identifiers, the range, whether the slot is required, and the pattern/allowable characters.

Beyond the relational tables, the PHES-ODM is maintained as a LinkML schema [29]—a single structured description of the model from which other formats can be generated. The validation toolkit already follows this approach, and the same schema can produce JSON Schema, SQL definitions, and graph-query interfaces as needs arise. This addresses the concern that each implementer might otherwise build their own “transport version” of the model by flattening the relational tables; any API or interface built from the schema starts from the same definition rather than diverging from it. LinkML in particular is used because it can generate JSON and YAML files easily and is an open and community-supported project, with a robust suite of support tools and documentation. The structure also automatically provides a globally unique URI or IRI to each class, slot, and enumeration, making all the structures in this format more FAIR-compliant data by default. An example LinkML slot definition for the samples table is shown in Figure 4b,c.

Separating entities also enables customizability. WWS programmes differ in scale, capacity, and approach; prescribing a single rigid structure would force local modifications that undermine interoperability. To address this, the PHES-ODM offers a minimal version containing only mandatory data and metadata fields, while the full model includes provisions for any additional items that larger programmes may wish to record. Figure 4a compares the entity relationship diagrams for the minimal structure (left) and the full version with all optional fields and reference tables (right).

The full PHES-ODM may appear complex, but the model operates in a field accustomed to large standards. LOINC, for example, contains over 70,000 codes, yet most clinics use only the top 2000–20,000 [22]. The same logic applies here: the PHES-ODM’s minimal template contains far fewer than 2000 parts. The aim has been to be robust while applying clear principles; the model’s customizability and minimal version are designed to make it approachable regardless of programme size.

### 2.2. Minimal Version of the PHES-ODM

**Figure 5:**
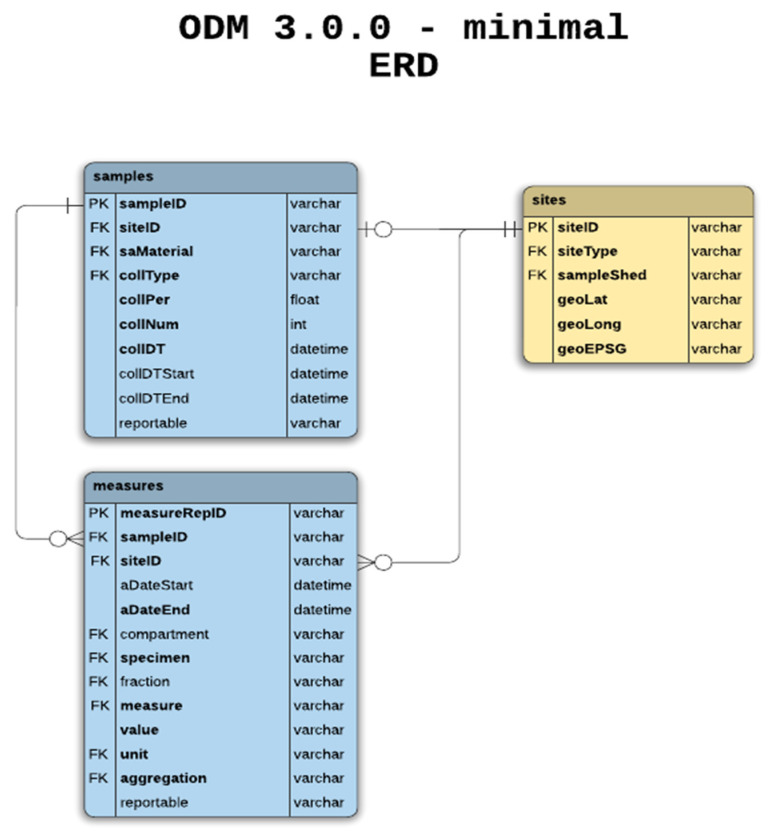
The minimal version of the PHES-ODM for version 3. It is composed of three tables: samples, measures, and sites. The information can be presented in one “wide” table or three separate tables linked by “keys”.

The minimal structure for the PHES-ODM includes only the mandatory tables and fields. It is purposely sparse and includes only what our user base have agreed are the essential fields. For WWS, we would like to always have more information and more metadata, but in situations of very limited resources, the information in this minimal structure is enough to record usable and reusable WWS information. An overview of the minimal structure can be found in Figure 5.

The samples table in the minimal template records only a sample identifier, a site identifier, the sample material, the type of collection, along with the collection period and number (for composite or continuous sampling), the date of collection (or start and end date) and whether the sample is reportable.

The sites table records only the identifier for the site, the type of building, and the sewage or environmental infrastructure from which collection occurs. This is recorded with the latitude and longitude for the site, and the EPSG coordinates.

The measures table records a unique identifier for each measure, with identifier linkages to the sample and the collection site. It also records the start and end dates for the measure analysis, the compartment of the measure (water, air, surface, or human), the specimen of the measure (site, sample, population, or polygon), the fraction of the wastewater being analyzed (only for the water compartment—solid, liquid, mixed, dry mixed, or wet mixed), the actual type of measure being recorded (e.g., population size, COVID N1 gene region, etc.) along with the recorded value for that measure, the unit and aggregation, and the indicator of reportability.

### 2.3. Model Governance and Future Transitions

Currently, the PHES-ODM operates with a centralized governance model. The principal investigator for the project makes major decisions and manages the initiative within the host organization, the Ottawa Hospital Research Institute (OHRI). This style of governance is not unusual in similar projects; however, PHES-ODM aims to shift toward a structure that is less reliant on a single individual and better supports the project’s values of open science and international collaboration. At present, the governance framework includes support from the International Steering Group, which was formed to guide major decisions and champion global adoption of PHES-ODM, and the Core Users Working Group, which is actively engaged in model development and user feedback. The Steering Group, however, has not convened frequently or recently, and the Core Users Working Group has absorbed many of its intended roles.

Moving forward, a governance plan has been drafted, proposing four main entities to balance strategic priorities with day-to-day operations and development support: the Maintainer & Scientific Advisor, which is the principal investigator role; the Executive Committee, which is a small team to oversee strategic goals, vision, and values; the International Steering Group, revived to convene a broader coalition of experts and stakeholders tasked with guiding major project decisions and promoting PHES-ODM’s integration into global environmental health efforts; and the International Working Groups, which are smaller, more focused groups that will continue to meet in regional clusters (e.g., the Pacific and Asia, North America and Europe) or based on thematic issues (e.g., data dictionary development, software testing). Once the executive committee convenes for its inaugural meeting, this governance proposal will be approved and then made publicly available.

This model will be preserved for the next three to four years of transition, after which the governance structure will be reassessed. The intention is to use this period to broaden representation and refine processes, so that a shift can be made to a more inclusive, internationally diverse board-style governance model. The possibility of shifting the governance and management of the model to a more permanent institution is also a possibility. This level of planning is important to avoid project “orphaning”, and to ensure project anchoring.

### 2.4. Audience and Aims for WWS Data

WWS programmes are, by their very nature, complex—they require collaboration between engineers, biotechnologists, policymakers, and public health professionals. The programmes require that all these groups be able to communicate effectively with one another. What is being communicated between these groups is often domain-specialized knowledge, making it hard to understand each other’s work or share a single database. Furthermore, different users and analysts may want different things from the data. By organizing the data around entities, we focus and streamline data entry by work domain. The model needs to include features to improve the usability of the data by various actors.

The target audience for the PHES-ODM is thus all these groups: engineers, biotechnologists, policymakers, and public health professionals. The model also intends to serve analysts—whether primary or secondary—who work with WWS data.

This complexity makes for a complex model. The PHES-ODM is very large and can be intimidating to new users. This is a major limitation of the model. To address this, we are committed to making our documentation as robust as possible. The discussion boards [30], walkthrough videos [31], and in-depth documentation [32] all help to improve the usability of the model. We are also working with large-language models and other artificial intelligence to develop assistants and notebooks to make it even easier to use.

While we have an entity relationship diagram (ERD) and a SQL definition for a database that uses the model, these more sophisticated pieces of programmatic infrastructure are not required to manage the model. The model was developed with an eye to provide ease of use. While this has not always been possible to maintain, the structure still strives for broad usability and can be managed as a Microsoft Excel file, or a series of CSV files. This ensures that even labs without access to SQL or other database management programmes can still use the model. For things like sharing or querying the data, the tools for sharing and validation can help with functions that might otherwise be difficult to manage outside of proprietary programmes. A version of the model is also available in YAML format using the LinkML [29] language. This way, users can bring the structure to whatever database management software they prefer.

### 2.5. WWS Data Standardization and Interoperability

The FAIR data principles—Findable, Accessible, Interoperable, and Reusable [12]—are among the most widely cited frameworks for data standards and data models. Other groups have tried to expand on these principles, either by adding layers of interoperability [33], or by expanding the principles to encompass other, more human-centric criteria, such as cognitive interoperability [34].

The European Open Science Cloud (EOSC) Interoperability Framework [33] specifically posits interoperability as having four layers, specifically: technical interoperability, meaning that Information Technology (IT) systems have completely understood interfaces and are able to work with other IT systems without restrictions; semantic interoperability, which ensures that the format and meaning of data is preserved and understood throughout exchanges between parties; organizational interoperability, meaning organizations align their business processes and responsibilities to achieve shared goals; and legal interoperability, which ensures that organizations can work together even when operating under different legal frameworks. Vogt’s additional data principles centre largely around ensuring data are cognitively interoperable. That is, data must be understandable and usable by humans, not only machines [34].

Researchers examining secondary use of population data—the situation facing most WWS analysts who are not the primary data generators—highlight the importance of robust metadata that makes clear the scope, limitations, and transformations already applied to the data, alongside a comprehensive data dictionary that defines variables and supports validity assessment [35]. Data standards, and the data dictionaries that define them, improve interoperability by enabling users to structure data and metadata in technically and semantically consistent ways. Yet the development of standards is not without problems. Some are proprietary, limiting adoption. Even among open standards, overlapping items are not always interoperable—open standards may be necessary for health data interoperability, but they are not sufficient on their own [36]. Across domains, data standards fall short of their interoperability goals when they do not actively prioritize interoperability with one another [37]. The solution is not a single, universal standard, but rather several major standards that can interoperate. Building on that line of reasoning, sound structural principles make for a good data standard, but so does working with other standards in the field. The PHES-ODM seeks to apply the interoperability principles outlined above while also working with other major WWS data standards to ensure data can flow between them (an overview comparing the PHES-ODM with other major WWS data standards is provided in Table 2). Interoperability offers tangible benefits—greater data access, more usable data, improved efficiency, and enhanced collaboration [38]. In public health surveillance, these benefits translate into lives saved.

Mapping between the PHES-ODM and NWSS has been particularly close: version 1 of the PHES-ODM was effectively co-developed with the designer of NWSS, with extensive discussion between the two groups during the early pandemic-era model design. NWSS subsequently formed the US CDC’s reporting framework, while the PHES-ODM continued to evolve to address a wider range of countries, users, and use cases; structural coverage is high, and the PHES-ODM contains all NWSS concepts. Mapping to PHA4GE has required more parsing of free-text fields, which the PHES-ODM team has addressed by developing scripts that map free-text content to controlled enumeration values. (Version 1 of the PHES-ODM included free-text fields with the explicit plan of using them to develop enumeration values, an approach we have continued and expanded.) A formal case study with quantitative loss and error rates was not feasible in this revision due to data-sharing constraints with the originating bodies, but we are open to a deeper analysis if it would be useful.

### 2.6. Semantic Integration and Shared Formatting

How data are formatted can impact useability and shareability. For example, representing date information in “year-month-day” vs. “day-month-year”, etc., can make it hard to combine data from different sources. To resolve this issue, the PHES-ODM tries to use International Standards Organization (ISO)’s guidance, where available, to avoid ambiguities. For dates, all dates are required to be in ISO-8601 format [39]. Enforcement for this structure is managed through the PHES-ODM’s validation library [40]. In a similar vein, the enumeration values for countries in the “addresses” table are all reported using the ISO 3166-1 alpha-2 country code [41], while regions are reported using the ISO 3166-2 code for country sub-domain [41]. To make the use of these codes easier, we have two reference tables included in the full model: one to manage the ISO codes for countries, and the other to manage the ISO codes for regions. The Excel templates have drop-down lists for inputting these items, and the validation tool makes sure the entries are valid.

Another method to ensure that even data recorded in other formats or models can still use the PHES-ODM is to partially adopt controlled vocabularies, such as ontologies. Ontologies for wastewater surveillance and public health interventions specifically can be quite sparse, so for certain categorical items, the PHES-ODM links to ontology references from ENVO [42], GenEpiO [43], and NCIT [44]. ENVO items help describe areas for sampling and sample collection in universal ways, while GenEpiO and NCIT have been adopted to describe more biological, laboratory, and surveillance items. These ontologies are not recreated within the PHES-ODM, but rather, the model’s data dictionary includes references to ontology entities to provide greater clarity around concept definition and overlap between data models. Using these kinds of controlled vocabularies helps to map between other ontology-using models, such as the PHA4GE data model.

Not everything within the PHES-ODM has an external linkage to an ontology entity, however, and this is for several reasons. The model was developed quickly, at least initially, for use by laboratories and surveillance teams. The need to have a shared concept defined and codified quickly was important, and integrating ontology references for each new item was not always prudent. The space of wastewater surveillance, too, is still somewhat liminal to ontologies and many items do not have ontological mappings available, or at least not ones that make sense for their domains. Other major data dictionaries and models for wastewater surveillance, such as NORMAN SCORE, W-SPHERE, USCDC’s NWSS, and AMELAG do not have any ontology references built into their models. Mapping between these models and the PHES-ODM—and the bulk of data mapping generally—thus relies on semantic mapping of concepts. There are many challenges with this approach, namely that how data model entities are defined, how they are used, and how they are interpreted by different groups and individuals can all be slightly different, though to some degree, these interpretational differences persist among ontology definitions as well [45]. To resolve some of these issues, there are levels of specificity within PHES-ODM enumeration values, so that in the absence of additional context, a higher-level category can be recorded. For example, samples can be collected from industrial wastewater treatment plants, municipal wastewater treatment plants for combined sewage, or municipal wastewater treatment plants for sanitary sewage. In the absence of additional details, this can all be recorded as simply a “wastewater treatment plant” generally. The definitions the PHES-ODM uses attempt to leverage this hierarchy of specificity, similar to classes and subclasses in ontologies, and to employ definitions that are clear and descriptive. Often, the definitions and categories, like those used for the “public health actions” table, are drafted in collaboration with users from various levels of WWS programmes in an attempt to ensure robust coverage of concepts in ways that are understandable to the userbase, and occasionally adapt definitions based on how similar or overlapping concepts are described in other models to ensure stronger semantic coverage.

A particular form of this challenge is syntactic alignment: the same term can mean different things in different programmes. The reviewer’s example of “raw wastewater” defined as influent in some programmes and as post-grit-chamber wastewater in others is one the PHES-ODM addresses structurally rather than by terminological convention. The model separates site type (the location, e.g., wastewater treatment plant, in the site Type enumeration) from sample material (the substance and its position in any treatment process, in the sample Material enumeration). A user recording a sample at a wastewater treatment plant, therefore, must specify which raw wastewater they mean—“rawWW” (raw sewage at the site, before in-plant treatment) or “pstGrit” (after grit removal at the headworks)—eliminating the ambiguity by structural decomposition rather than by relying on shared interpretation of a single label.

Geographic place identifiers such as Geonames are not enforced at the country/region level—those use ISO 3166—but can be attached at the polygon level for individual catchment areas, sites, or sub-regions, preserving flexibility where finer-grained location identifiers are useful.

A natural extension of this approach is to maintain the mappings between PHES-ODM and other models—PHA4GE, NWSS, AMELAG—as data themselves rather than only as documentation. Emerging standards such as the Simple Standard for Sharing Ontological Mappings (SSSOM) [46] directly support this, and the LinkML schema already allows links to external ontology terms within each slot definition. Together, these positions PHES-ODM less as a fixed dictionary and more as a connecting layer between WWS data formats.

### 2.7. Lessons from Other Data Standards and Models

The field of medical data models and standards is not new, and there are lessons to be learned from their example. Take for example the Health Level Seven International (HL7) Fast Health interoperability Resources (FHIR) tools [47] and the Observational Medical Outcomes Partnership (OMOP) Common Data Model (CDM) developed by the Observational Health Data Sciences and Informatics (OHDSI) programme [48]. Both are data models used to improve the use and sharing of clinical medical data. Both models have a purpose similar to that of the PHES-ODM; they seek to make it easier to share and aggregate data and to use and analyze it more quickly and easily. The differences are that FHIR and the OMOP-CDM exist for clinical patient data, rather than for public health or environmental surveillance data. Both models can boast strong adoption since their founding, in part due to changing laws on health data and support from major technology corporations in the private sector [49]. Pressure from health system payers (i.e., insurance companies, governments) has also pushed for greater adoption.

The existence of two widely adopted standards, and other less adopted ones, again highlights that having a single standard is often not plausible in practice. But within the health data ecosystem, many tools already exist to move data between these formats [50]. There are many lessons to be learned from these examples, such as the importance of community buy-in, demonstrated utility and ease, the importance of governance, and ultimately that large-scale adoption of standards is possible [51].

The PHES-ODM’s development included a deliberate review of established health-data standards alongside its practical origins. The model began with different WWS users comparing spreadsheets at the start of the COVID-19 pandemic to find common ground; the project’s steering and operations committees subsequently reviewed FHIR, the OMOP-CDM, LOINC, and other public health dictionaries against the WWS use case. None fit the wastewater domain directly, but conceptually LOINC—as a shared dictionary for observations across many laboratories—was the closest precedent. Lessons from LOINC’s adoption (stable identifiers, sustained governance, and accommodation of users at varying levels of technical capacity) have shaped how the PHES-ODM evolves, and the model is positioned as a domain-specific complement to these standards rather than a substitute.

### 2.8. Structural Solutions for Data Challenges and Interoperability

Data standards and dictionaries are designed to ensure that data are generated, used, and shared easily, but problems persist due to structural issues in the data standards, or user error in their application. The literature on issues encountered in population and public health data is strong, and the issues persist across research disciplines [35,39,41]. Of particular concern are challenges around: ambiguous data definitions; a lack of contextual information (methods applied, standardization of data definitions, location data); lack of clarity on the temporality of the data; ambiguous data revisions, or no capacity to perform data revisions; unreported differences in the level of spatial and temporal resolution across the data; uncertain data quality; ambiguous ownership, credit, and licensing of data; and difficulty in databases and data infrastructure around balancing robustness against ease of use.

The super-issue that underlies most of these problems is ambiguity. With data, and secondary use of data, we do not know what we do not know. Clearly recording maximum information is the only solution for avoiding misuse and misrepresentation of population and surveillance data. The issue exists throughout the lifecycle of the data, and if data collectors are unaware of what context is important, it will not get recorded. The PHES-ODM has thus tried to address these challenges structurally, by explicitly including provisions to counter these important ambiguities:

- Data dictionaries and ontology integration to counter ambiguous data definitions.
- Protocols and calculations data tables to counter a lack of contextual information.
- Data relevancy periods to clarify data temporality.
- “Last edited” and “notes” fields for tracking data corrections transparently.
- A “site level” and “specimen” field for tracking spatial resolution of the data.
- “Reportable” and “quality flag” fields for recording measure quality issues; a validation library to improve data quality issues.
- “License“ fields connected to datasets and measures to ensure responsible and legal use.
- Documentation and online community resources to balance ease of use against a robust model.

The other barrier to interoperability through data standards is a lack of adoption. Many researchers argue for the importance of data standards and interoperable data, but few implement standards in their work. Within the GLOWACON technical working group on data, a survey of the membership indicated that the majority thought interoperability and data standards were important, but almost none used a data standard themselves. This is a complex problem that is related to a myriad of factors (lack of awareness, lack of implementation tools, lack of time), and the PHES-ODM is trying to address them all through:

- Outreach and extensive documentation.
- Building tools with built-for-purpose interfaces, including data parsers and validators.
- Ready-to-use templates for common use cases, and video documentation to support their uptake.
- Adoption incentives.

**Table 2:** An overview of the most commonly referenced data models for wastewater and/or environmental surveillance, and a comparison between them. This table is based on Therrien et al.’s Table 1 [14] but is expanded and updated to include additional categories and reflect the current landscape of environmental public health data models.

Feature	PHES-ODM	NORMAN SCORE	W-SPHERE	NWSS	PHA4GE	AMELAG	MIxS
Reference	Manuel et al., 2021 [13]; Therrien et al., 2024 [14]	NORMAN Network, 2020 [52]	Global Water Pathogens Project, 2020 [53]	USCDC [16]	Griffiths et al., 2022 [54]; Paull et al., 2025 [55]	RKI, 2025 [56]; RKI & UBA, 2026 [57]	Genomic Standards Consortium [58]
Intended Audience	WWS practitioners (public health authorities, Engineers in WWS)	Ecotoxicologists, SARS-CoV-2 template for WBE practitioners	WWS practitioners	WWS practitioners	Environmental Genomics	WWS practitioners	Environmental Genomics
Documentation language	Narrative documentation: English; database definitions and data dictionary: English, French, Spanish, Portuguese	English	English	English	English	German, English	English
Public data dictionary of headers and tables	Yes	No	Yes	No (outdated version available from USCDC archive)	Yes	Yes	Yes
Public data dictionary of values	Yes	No	No	No (outdated version available from USCDC archive)	Yes	No	Yes
Database structure type	Relational database	Flat-file database	Flat-file database	Flat-file database	Flat-file database	Flat-file database	Flat-file database
Public database definition	Yes	Yes	No	No	Yes	Yes	No
Public data conversion tools	Yes	No	No	No	Yes	No	No
Public data validation tools	Dictionary, software tool, templates, validation rules schema	Template	Template	None	Dictionary, software tool, templates	Dictionary, validation rules schema	Dictionary, software tool
Public data sharing infrastructure	Python library, explicit measure and dataset licensing, external dataset linkages	No	High-level dashboard	High-level dashboard	Software tool (DataHarmonizer v1.6.5)	Dashboard, Zenodo publication of data	Many repositories require adherence to this standard to share
Public data collection templates	Yes	Yes	Yes	No	Yes	No	No
Governance and development	Open source	Inter-institutional	Internal	Internal	Open source	Internal	Open source
Model license	CC-BY4	Not found for the model, but the template is open access	Not found	Not found	CC-BY4	CC-BY4	CC-BY4
Clear channels for user feedback	GitHub issues, Discourse discussion board, email maintainers	Email maintainers	Email maintainers	Email maintainers	GitHub issues, email maintainers	Email maintainers	GitHub issues, email maintainers
Rights management	Element level (any row, header or combination)	Dataset level	Dataset level	Dataset level	Dataset level	Dataset level	Dataset level
Environmental compartments	Various	Various, but only wastewater in the template	Wastewater	Wastewater	Various	Wastewater	Various
Pathogen measurement	Any pathogen in the dictionary (Multiple+)	Yes, but only SARS-CoV-2 in template	SARS-CoV-2-specific	Multiple	Multiple	Multiple	Multiple
Detailed protocol recording and linkage	Yes	No	No	No	No	No	No
Detailed sample relationship records	Yes	No	No	No	No	No	No
Measurement methods	Yes	Yes, but only PCR and sequencing-specific in the template	PCR and sequencing-specific	PCR and sequencing-specific	Sequencing-specific	Not found	Sequencing-specific
In-sample measurements	Any measure in the dictionary	Water quality, but only PCR in the template	PCR and sequencing	PCR and sequencing, pH, Conductivity, TSS	PCR and sequencing, water quality	PCR and sequencing, pH, temperature	PCR and sequencing, water quality
Collection site information	Yes	Yes	Yes	Yes	Yes	Yes	WWTP infrastructure details
On-site measurements	Any measure in the dictionary (expandable)	Flow, weather, COD, TSS, NH_4_^+^-N, water temperature	Flow	Flow, water temperature	Flow, weather, COD, TSS, NH_4_^+^-N, water temperature, conductivity, pH, contamination	Flow, pH, temperature	COD, TSS, NH_4_^+^-N, phosphate, salinity,
Population count	Served by site, or within a geographic region (polygon)	Served by site	Served by site	Served by site	Served by site	Served by site	No
Sewer network information	Possible to record details as measures in the dictionary	No	No	Average wastewater travel time, industrial input, stormwater input	Upstream activity and treatment	No	Industrial input, reactor type, sludge retention time
Sample and sampling method	Yes	Yes	Yes	Yes	Yes	No	No
Used by a national/ supranational WWS programme	Yes	No	No	Yes	No	Yes	No
Records provenance and transformation steps	Yes	No	No	No	Yes (accession IDs for reference sequences; libraries and processing software)	Yes (reports viral load, flow-standardized viral load, and predicted viral load)	Yes (libraries and processing software)
Genomic repository linkages	Yes	No	No	No	Yes	No	Yes
Population health data	Any measure in the dictionary (aggregate health data—population level)	SARS-CoV-2 prevalence	No	No	No	No	No
Ontology integration	Limited	No	No	No	Yes	No	Yes
Interoperable with at least one other major dictionary using public tools	Yes (PHA4GE, NWSS)	No	No	Yes (PHES-ODM; managed by PHES-ODM)	Yes (PHES-ODM; managed by PHES-ODM)	No	No

## 3. Results and Discussion

### 3.1. Addressing the Audience: Public Health Surveillance

With WWS, we are surveilling the environment for evidence of infection and threats to human health. As a disease surveillance system, it exists under the umbrella of public health. The data generation, however, comes from sites and sampling that exist outside of the typical (i.e., clinical) setting for public health surveillance. Most of the research, testing, and programmes, at least in Ontario, are or were run by engineering and environmental science laboratories [59]. This ensures relevant contextual data around sites, samples, and wastewater processing are collected, and that the best information about the presence of pathogens and other threats to human health are being extracted from the complex sample matrix that is wastewater. This context is unfamiliar to public health practitioners, however, and translation across each discipline’s dialect is often required.

Version 1 of the PHES-ODM was quickly developed with partners and health authorities to create an open data standard at the height of the COVID-19 pandemic response. The infrastructure to facilitate communication between engineers and public health teams at that point was including provisions to store case, hospitalization, and death data in the same dataset as WWS data. As the programme developed, however, needs changed. As public health departments started predictive modelling projects, alerting them to which measures to use, or which not to use, became very important. In version 2 of the PHES-ODM, more robust quality flagging was added for measures and samples. More importantly for public health departments and analysts, however, was the new “severity” attribute to alert them on how important a quality issue was. A binary (“TRUE” or “FALSE”) “reportable” field was also added to both the “measures” and “samples” tables so analysts can tell immediately whether the data is dependable. The data dictionary (the “parts” table) is also available to provide additional guidance on the meaning of terms.

#### The Public Health Actions Table

Building on previous work, version 3 of the PHES-ODM maintained the original features to support public health departments in understanding WWS data or expanded them. The public health aspect of the data has also been made even more explicit by adding the optional “Public Health Actions” table to the model. The purpose of the “Public Health Actions” table is to link health-related policy actions to the measures that inform and/or result from them.

**Figure 6:**
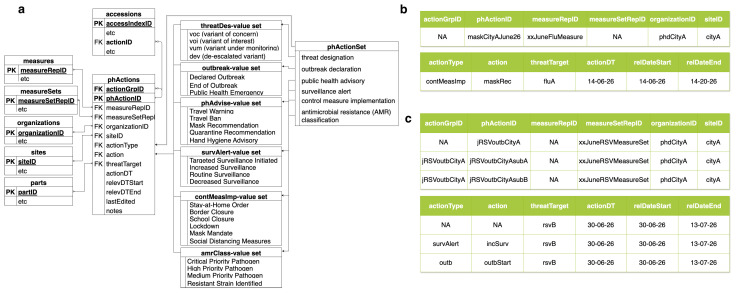
(**a**) Entity relationship diagram for how the public health actions table is integrated into the PHES-ODM model. The figure shows how the other data tables (namely: “measures”, “measureSets”, “organizations”, “sites”, and “parts”) integrate into the “phActions” table through referencing those tables’ primary keys as foreign keys. It shows how actions recorded in the “phActions” table can be linked to external public health data through referencing the phActionID as a foreign key in the “accessions” table. Lastly, it shows the enumerated values for the “actionType” and “action” fields, grouped by action type in the tables on the right. (**b**) Example data entry for a single action in the phActions table. The example uses fictional data from a public health department (phd) for City A issuing a masking recommendation (“maskRec”) as an infection control measure (“contMeasImp”) for influenza A virus (“fluA”). (**c**) Example data entry for a group or multi-pronged action in the phActions table. The example uses fictional data from a public health department (phd) for City A declaring the start of an outbreak (“outbStart”) as part of an outbreak alert (“outb”) for respiratory syncytial virus B (“rsvB”) and increasing surveillance of that same pathogen (“incSurv”) as part of a surveillance alert (“survAlert”).

The table and the values for its enumerated fields can be found in Figure 6a. “phActionID” serves as the primary key for the table and is a unique identifier for each row or action taken. If multiple actions are being taken at the same time, a second “phActionID” can be generated as an umbrella action ID, and the “actionType” and “action” fields left blank. The actual entries for each of the related actions will then use the “phActionID” of this partially blank row as the action group ID (“actionGrpID”) (see Figure 6c for an example). The measures, or group of measures, related to the public health action are linked to it by their IDs in the “measureRepID” or the “measureSetRepID” field, where they act as FKs. Currently, the causal relationship between measures and actions is left ambiguous; the fact that there is a relationship is specified, but it is not made clear if the action is a result of the linked measures, or whether the measures are a result of the action. The organization responsible for the public health action is linked using the “organizationID” as an FK from the “organizations” table. The site, if relevant (particularly for a hospital or clinical setting, for example) can be linked, using the “siteID” as an FK from the “sites” table. The “actionType” field serves as a high-level general organizer for the type of action being undertaken, while the “action” field adds more detail. “threatTarget” is the pathogen being targeted by the action, and it is populated by “measurement” part types from the parts table (i.e., items like “sarsCov2”, “fluA”, etc.). In instances with multiple targets, each pathogen will need an individual row, and they can be grouped using “actionGrpID”. The date and time of the public health action (“actionDT”) is recorded in this table, along with a relevance start and end date (“relDateStart” and “relDateEnd”) to mark the projected period of activity for a given action. Finally, the “lastEdited” field and the “notes” field record when (if ever) a row in the table was updated, and any additional details about that action, respectively.

One of the first uses for this table was for threat designation, to report whether a variant was a variant of concern or delisted at the time of reporting. Building from there, we wished to also record other types of actions taken, drawing from experiences and policy interventions used in the COVID-19 pandemic, and for influenza and other more endemic but important public health threats. We sought initially to use existing standards and looked at adopting elements from the International Classification of Health Interventions (ICHI), from the World Health Organization Family of International Classifications (WHO-FIC) [60]. While there are other medical intervention ontologies and standards, the public health elements in them are often very thin. Other scans of the literature pointed instead toward behaviour and behaviour-change ontologies. Ultimately, ICHI, and other ontologies we looked at were not detailed enough for what we wanted. For example, there are items such as “Population public health surveillance”, “Population quarantine measures”, and “Population alert” in ICHI, but there is no level to distinguish between recommendations vs. mandates, or to determine changes in these interventions (i.e., increasing, initiating, decreasing, etc.). To develop the current list, we drew on our experience as public health physicians and practitioners and developed the list in collaboration with partners in public health institutions. We think the absence of a public health ontology is a large gap in the field, and while we are not equipped to be able to draft such an ontology ourselves, we do hope someone will take up this banner in the near future.

Figure 6b,c walk through the following examples for using the table: a public health department (phd) for City A issues a masking recommendation (“maskRec”) as an infection control measure (“contMeasImp”) for influenza A virus (“fluA”); and that same public health department in that same city declares the start of an outbreak (“outbStart”) as part of an outbreak alert (“outb”) for respiratory syncytial virus B (“rsvB”), increasing surveillance of the pathogen (“incSurv”) as part of a surveillance alert (“survAlert”).

### 3.2. Addressing the Audience: Data Analysts

**Figure 7:**
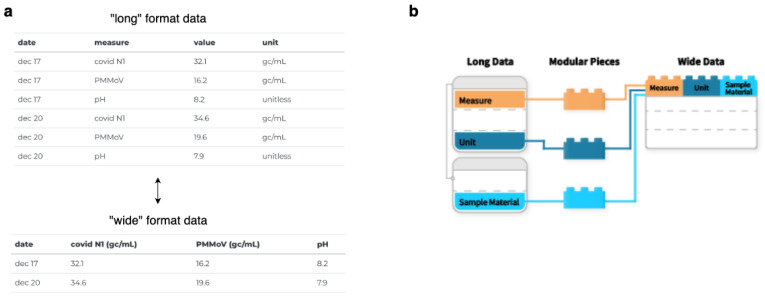
(**a**) Example of the same measurement data in “long” vs “wide” formatting. The example shows a series of measures for two dates in the “long” format, with one measure per row. It then shows that same data in the “wide” format, with one row per date, but multiple columns now for each measure. (**b**) Illustration of the modularity of the relational PHES-ODM data structure in practice. This figure shows how different headers or attributes from different tables in the PHES-ODM structure can be selected and combined into a single table for more customized templates for data entry and/or analysis.

Across the data lifecycle, users need data organized differently during analysis than when stored in a database. Moving between these two formats, however, may introduce errors and cause friction. To support reproducible data transformation and avoid errors, the PHES-ODM supports a “wide” data format. For our purposes here, we will define “long” data as a format where one row represents a single entity, while “wide” data uses columns to store information about multiple measures or entities per row, usually for a shared entity like a date or a site. An example comparison is shown in Figure 7a, where in the “long” format the measures for a date have their own rows, and in the “wide” format each date is a row, with additional columns for each measure. The standard PHES-ODM format is the “long” data format, which is ideal for storage and database scalability, and ensures that the data is very machine-actionable. For larger analyses and faster data entry, however, the “wide” format is often preferred. While convenient for quick data entry, the compactness of wide tables leaves little room for metadata beyond the column header.

To support analysts, a standardized model for naming columns is essential for ensuring that wide columns have known names, allowing for analytic code to be shared. A “wideNames” reference table is now in the model to record currently active wide column names in use in PHES-ODM templates. Wide names combine dictionary terms to form a column header that effectively encodes metadata that would occupy fields in a long table. The Excel template that includes the wideNames table also has the functionality to help users generate new compliant wide names based on the data and metadata they wish to collect, allowing for interoperable wide–long transformation. The rules for generating valid, machine-actionable names are available in the model’s documentation [61].

To our knowledge, the PHES-ODM is the only data model that has explicitly tackled the long-versus-wide problem in this way. In practice, many WWS programmes—including most national programmes during the COVID-19 pandemic—work with wide-format data, often in Excel; data scientists and database designers prefer long-format relational data. The PHES-ODM accommodates both by maintaining the relational schema as the canonical long form and generating wide variable names from it. Each part identifier in the dictionary is constructed from alphanumeric characters with no leading number, so identifiers can serve as valid variable headers in any analytic tool. Concatenating these identifiers (e.g., site, sample, target, unit) forms a structured wide-column name that is both unique and reversible: wide tables can be transformed back into the long form without loss of information. This bridges the practical preference for wide spreadsheets with the relational rigour required for interoperable storage—directly supporting the model’s broader aim of being both robust and easy to use.

The generation of wide names and their use in templates showcases the modularity of the PHES-ODM model by facilitating template generation customized to different programme needs. The model is larger to be robust and to accommodate as much data for as wide an array of potential users as possible. The robustness is meant to support compliance to the model and avoid having users modify the model locally to respond to a need (creating problems for interoperability). Most fields in the model, however, are optional. The full model can be compared to a restaurant menu or a bucket of Lego bricks; all the items are available to you in their standard format, and it is up to you (and the needs of your programme) to determine what you select, adopt, and use. This applies to both the long and wide versions of the model. An illustration of this kind of modularity is shown in Figure 7b.

While modularity is a strength of the model in addressing the diverse needs of different programmes, we are also aware that this customizability may place a cognitive burden on new users. In determining which fields to use and how to use them, there is much to consider and understand. To this end, we have pre-made templates to facilitate adoption, as well as documentation and video walkthroughs to support users. While the pre-made templates, documentation [32], and walkthroughs [31] can only take users so far, the discussion board for the PHES-ODM [30] offers a place to ask questions and get support from the model’s developers and other users. All data models and standards will place some cognitive burden on users, though steps can be taken to ease adoption and use.

### 3.3. Addressing Standardization Challenges: Data Mapping and Interoperability

**Figure 8:**
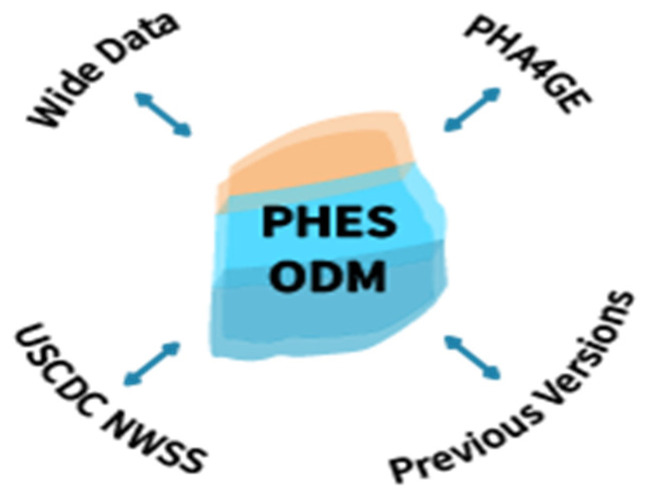
The PHES-ODM as an interoperability pathway and Rosetta Stone for global WWS data.

As mentioned above, achieving a single, unified data standard for WWS is unrealistic. Developing open standards, like the PHES-ODM, is part of the solution, but is insufficient. There is a proliferation of non-interoperable systems within and outside the field of WWS. The developers of standards and models need to prioritize interoperability and to work to build tools to interface with other systems and standards [37]. To this end, the PHES-ODM aims to serve as a Rosetta Stone (Figure 8) between other WWS data standards and models. Currently, data in the PHA4GE data format [54,55], the USCDC NWSS data format [16], as well as previous versions of the PHES-ODM can all be mapped into version 3 of our model using tools developed by our team [62].

The easiest parts of interoperability are the basic objects, such as categorical inputs, unique identifiers, and date fields—perhaps in part because these objects tend to be non-ambiguous. The main struggle here is to ensure data being brought into the PHES-ODM format have destination fields that match. Ensuring that the PHES-ODM has overlap with other WWS data models has, for example, led to a proliferation of date-type fields. Because different labs and standards record date information in different ways, version 3 of the PHES-ODM now includes alternative structures for recording dates and times. These alternatives include recording the epidemiological week [63], including the start date and year of that epidemiological week; and recording the date with a categorical generalization of when the sample was collected (i.e., morning, afternoon, evening, and night). Beyond these easier targets, metadata can be difficult to match across standards due to different scales of recording and measurement, structural differences, or imperfect semantic overlap. This is not a novel problem, and natural language translation has worked around imperfect translation since time immemorial. Heterogeneity between data models, even when interoperability tools are available, still run the risk of information loss or truncation during the mapping process. Because the incoming data was not recorded in the destination format, it may also be missing otherwise mandatory fields. Problems can arise when imperfect translation is left ambiguous or unexplained. To help explain possible irregularities generated by mapped and transformed data, version 3 of the PHES-ODM has a field in the “datasets” table for recording the “originalFormat” of the data, also supporting greater data transparency.

**Figure 9:**
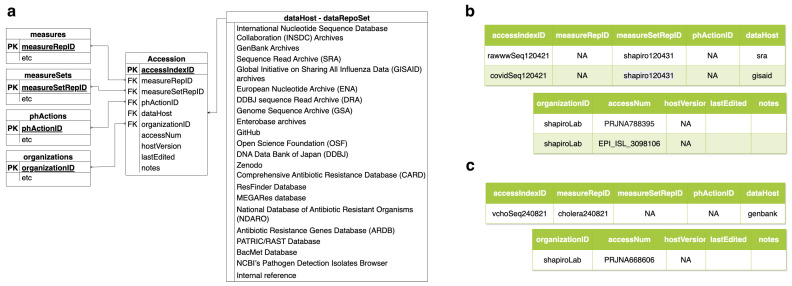
(**a**) Entity relationship diagram for how the accessions table is integrated into the PHES-ODM. The figure shows how the other data tables (namely: “measures”, “measureSets”, “phActions”, and “organizations) integrate into the “accessions” table through referencing those tables’ primary keys as foreign keys. It shows the possible categorical inputs for the “dataHost” fields. (**b**) Example data entry for two linkages to a measure set in the accessions table. A toy example generated based on N’Guessan et al. [65]; the example shows two accessions IDs linked to one measure set, referencing the SRA database the raw wastewater sequencing data (row 1), and viral genomes from clinical samples in GISAID (row 2) for the Shapiro lab, with arbitrarily invented IDs. (**c**) Example data entry for a single external reference in the accessions table. The example shows linkages to a single measure, for metagenomic sequence data that are available in GenBank for the Shapiro lab based on Levade et al. [64], with arbitrarily invented IDs.

Linking out to the dataset in its original format is also possible thanks to version 3’s new optional “accessions” table. This table is designed to link external data, whether that be original data sources; large data that cannot be stored well outside its context (i.e., sequencing data, GIS data); or public health data and dashboards. An entity relationship diagram of the table can be found in Figure 9a. The “accessionIndexID” is the PK for the table and is a unique identifier for each row. The “measureRepID” or “measureSetRepID” link as FKs to a single measure or sets of measures related to the external data being referenced. For example, if a measure is reporting the proportion of a given variant found in a sample of wastewater, that measure can be linked to the accessions to point out to the full sequencing data from that finding. The “phActionID” works like the measure identifiers, but links public health actions to external data on the details of that action. “dataHost” is a categorical variable for reporting to what repository the accession is pointing. For internal databases linking across different departments or sectors, an “internal reference” category is also available. The “organizationID” field links to the organization that is associated with the external data. For example, users may point out to a reference sequence generated by another group that was used to confirm their findings. The “accessNum” field is a free text field for reporting the accession number or ID for an entry in the “dataHost” repository. “accessNum” can also take a web address as an input. To report on different repository versions, where applicable, “hostVersion” can be used to record that data. Lastly, as with all PHES-ODM tables, the optional “lastEdited” field and “notes” fields record when data were last updated, and any other details, respectively.

Figure 9b,c provide a fictional example of how accession data might be recorded using examples constructed from references in two papers from the Shapiro lab [64,65]. Figure 9b shows two accessions linked to the same set of measures as an example, with one pulling the SRA database Bioproject accession for the raw wastewater sequencing data (Figure 9b, row 1), and the viral genomes from clinical samples in GISAID (Figure 9b, row 2). Figure 9c shows linkages to a single measure, for metagenomic sequence data that are available in GenBank.

### 3.4. Implementing Structural Solutions: Expanding Metadata and Their Context

The bulk of the PHES-ODM is metadata. The measures take up very few fields, while provisions for contextual information are numerous. This is in part due to the complexity of environmental surveillance, which requires extensive metadata to make the information found in measures useable. Ensuring that the data generated by these programmes are useable is also a core responsibility of WWS programmes, as a part of managing data ethically and upholding principles of data justice. This does not mean all data should be publicly available—while no exact number has been verified, and community shedding dynamics likely differ widely, some programmes are already withholding data about smaller sewersheds (<3000 people) on the grounds that they may not benefit from a large enough pool for the data to be anonymized [66,67]. This means that some WWS data are sensitive personal health data. For sites in large urban centres, however, this is not an issue. While Brown et al. state that the three things that matter in science are the data, the methods for data collection, and the logic that connects the former two to their conclusions [68], the greater the context and metadata provided, the greater is the logic to ground one’s conclusions.

Metadata fields added in version 3 of the PHES-ODM were primarily added at the request of users. Others were added to provide structural solutions to the common data problems mentioned in the methods Section 2.4, Structural Solutions for Data Challenges and Interoperability. The impetus for structural solutions is that discussion of data problems does not necessarily provide solutions; acknowledging a problem is not enough. Some of the structural solutions have been a part of the model across several versions, but their specific use will be discussed here. By building in specific metadata fields to eliminate these problems, the aim is to support users in generating better data.

#### 3.4.1. Implementing Structural Solutions: Data Definitions

Issues around ambiguous data definitions are alleviated by the data dictionary, which has been a part of all versions of the PHES-ODM. By providing explicit definitions and instructions for every field and value within the model, ambiguity is resolved. The use of controlled vocabularies, like ontologies, are also a great boon to ensuring shared definitions. For version 3 of the PHES-ODM, the ontology integration was expanded and will increase with successive releases. Some data definitions have expanded or shifted across version releases of the PHES-ODM. Using ontology integration helps to limit issues caused by these updates, but the model also includes changelogs between versions.

#### 3.4.2. Implementing Structural Solutions: Defining Temporality of Data

Temporality issues are another challenge addressed in the PHES-ODM. A feature of the model that has existed across multiple releases is the “lastEdited” field in all tables. In discussions with users, this field is overwhelmingly left blank or not included, largely because mistakes are caught early or revisions are seldom required. It is still something we include as an optional field to allow users to make changes when needed. This field allows users and data managers to update data entries to correct errors, while making the correction process transparent. A similar issue for temporality is recording a context window for data. For example, the measures of population size are generated from census data which are used for five years, or until the next census results are released. Editing the population counts for every census may change the denominator for past calculations and lead to erroneous conclusions. Providing a context window using relevance start and end dates (“relDateStart”, “relDateEnd”) to certain tables allows users and data generators to define the periods for which certain data apply.

Even with these functions, there is currently no explicit versioning or archive functionality for measures. Using the relevance start and end date fields, it is possible to look at shared information across rows (for example, measures of population size, for a specific site) and see some versioning that way. The model is built to be usable with only Microsoft Excel or CSV files, so more sophisticated versioning is difficult to employ, but remains under consideration for successive versions of the model.

The PHES-ODM’s temporality and versioning fields also reflect a less-discussed driver of the model: requirements that arise in commercial and government laboratory operations. Many laboratory information management systems (LIMS), and the legislation that governs them, require explicit logging of changes, corrections, and reporting breaks. The “lastEdited” field, the “reportable” flag, and related metadata fields exist in part to satisfy these requirements—one of several ways the PHES-ODM has been shaped by direct user requests rather than abstract design. These fields saw common use during the early phase of the pandemic, when laboratory methods were still being developed, and previously collected samples were sometimes re-analyzed as protocols matured; their use today appears less systematic, as discussed above.

#### 3.4.3. Implementing Structural Solutions: Contextualizing Data Quality

Data quality issues are a perennial problem in data work. As discussed above, some data quality concerns can be managed by using quality flags and the “reportable” field. This records quality issues at the sample, measure, or methodology level, along with an indicator of their severity. It also provides, via the “reportable” flag, a quick binary flag for whether a measure or sample should be included in calculations or reports. Reasons that a measure or sample might not be reportable largely concern processing. For example, a sample may not be reportable because it was stored improperly, spilled, or the label was lost. Measures may also be deemed unreportable for a of myriad reasons, such as potential inhibition/activation, plate-positive control trends, the measure being for research- or reference-only, or simply at the data owner’s request. Data entry or validity errors are, however, not possible to address or catch in this way. To resolve these issues, our group developed a validation tool using Python v3.12.4 [40,69]. This tool has detailed documentation and returns a report on data validity issues.

The PHES-ODM validation library [40] supports a range of automated checks: mandatory-field and data-type validation, numeric value-range checks (greater-than-max, less-than-min) for catching implausible values, controlled-vocabulary checks for category fields, duplicate-row detection, and missing-data scanning. The library is designed to be extended: users can register custom rules for their own programmes—for example, restricting site ID to a list of approved testing sites or enforcing project-specific naming patterns. The reviewer’s more specific concerns—temporal consistency rules and cross-parameter correlations—are not shipped as defaults, but the library’s extensibility supports them when users have the domain knowledge to specify the rules. An earlier separate tool, the Ontario Automatic Data Pipeline (developed in partnership with the Delatolla lab and other groups in the Ontario Wastewater Consortium), automated more of these domain checks for a single pathogen; heterogeneity in how different labs performed checks made a single shared tool hard to maintain, and complexity grows considerably across the many pathogens that the PHES-ODM now supports. Coherence checks specific to a programme’s protocols typically still require domain-aware validation run by the programme itself; whether common patterns of this kind should be elevated into shared default rules is an active question for future versions.

#### 3.4.4. Implementing Structural Solutions: Ownership and Licensing

Data ownership and legal interoperability is a major concern in research—particularly around concerns of “getting scooped” on data publications—and consideration of proper attributions and data provenance have been present since the inception of the model. Version 2 of the model included the “datasets” table to provide information on who generated the data. This table has numerous linkages to properly connect data to their sources. Version 3 builds on this by adding a “license” field to record the licensing of a dataset. A measure license (“measureLic”) field was also added to the measures table to record licensing at the level of individual measures if necessary. The suite of tools designed to support the PHES-ODM also includes a sharing tool [70] which uses an allow-list approach to automatically filter data for sharing.

There are, of course, limitations to this approach. Namely, that providing licensing information does not enforce adherence to that license. There are currently no technological solutions within the PHES-ODM to embargo data or enforce usage rights. Ultimately, there are limits to what we can achieve within a data model on this point. By making the licensing of items explicit, however, the rules for data and the repercussions for violating these rules are made explicit, and thus it is easier to explore recourse with intellectual property rights enforcement. Similarly, protecting data from small sewersheds (>3000 people) and Indigenous nations is important. The “reportable” flag, in combination with the license field, may be helpful in making sure these sensitive data are not shared. Particularly when using the PHES-ODM’s sharing library, the license and reportability fields are very useful for controlling what is shared. More robust approaches for data embargo and sensitive data management are under consideration for future versions of the model as well.

Beyond the license field itself, the PHES-ODM addresses sensitive data concerns through structural and governance mechanisms rather than statistical methods such as differential privacy. The license field has a clear limitation—providing licensing information does not enforce adherence—but making licensing explicit clarifies the rules and the consequences of violating them, which simplifies recourse through intellectual property rights enforcement. Three further mechanisms are particularly relevant for small or sensitive sewershed and Indigenous data. First, the model includes explicit sensitivity flags grounded in OCAP—the principles of Ownership, Control, Access, and Possession that govern First Nations data sovereignty in Canada—with provisions for racialized and otherwise sensitive populations alongside Indigenous data. Second, the PHES-ODM sharing library [70] allows users to define data-sharing schemas down to the data-element level, supporting multiple schemas per partner: distinguishing what data is shareable from what exists, and presenting the schema itself separately from the data. The “reportable” flag, in combination with the license field, is particularly useful for protecting data from small sewersheds (<3000 people) and Indigenous nations within these schemas. Third, data can be reported at the aggregate level, with version 3 adding explicit provisions for recording the transformations applied (moving averages, geographical aggregations, and other derivations). Together these mechanisms support the kind of selective disclosure that differential privacy aims to provide, while preserving data structure rather than introducing statistical noise. More robust approaches to data embargo and sensitive-data management remain under consideration for future versions.

#### 3.4.5. Implementing Structural Solutions: Data Treatments and the Calculations Table

**Figure 10:**
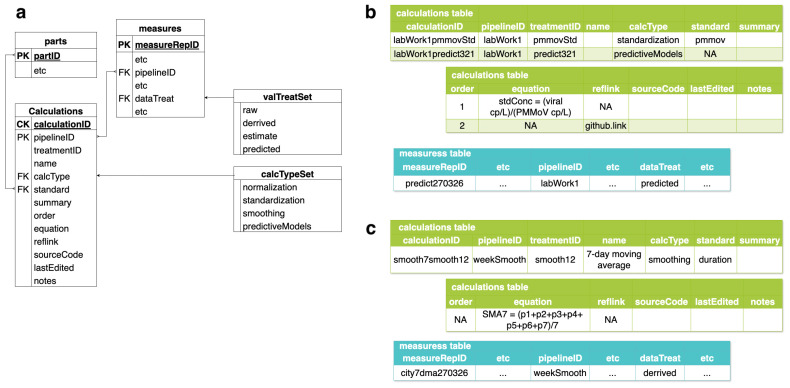
(**a**) Entity relationship diagram for how the calculations table is integrated into the PHES-ODM. The figure shows how the values for the “standard” field are pulled from the parts tables, and how the calculations table is linked to the measures table. It shows the enumeration values for the “calcType” field, and for the new “dataTreat” field added to the measures table. (**b**) Example data entry for a two-step data pipeline in the calculations table. For recording an estimated measure in the “measures” table, generated by a predictive algorithm that uses WWS data standardized to measures of pepper mild mottle virus (PMMoV), the predicted measure is recorded in the “measures” table, with a “predicted” value in the “dataTreat” field for the measures table. In the “calculations” table, the standardization of the WWS data to PMMoV would be recorded as one data treatment, with the “calcType” field recording “standardization” and the “standard” field recording “pmmov”. The “order” field in this row would record “1”, as it is the first step in the pipeline. The predictive algorithm would be recorded as a second treatment, with the “calcType” of “predictiveModel”, and the “standard” left blank or “NA”. The “order” in this row would be “2” as it is the second treatment in the pipeline. Both rows would share the same “pipelineID”. (**c**) Example data entry for a single-treatment pipeline in the calculations table. If a 7-day moving average calculation is done to smooth WWS data, the “dataTreat” field for the linked measure in the “measures” table would have a “derived” value. In the “calculations” table, the “calcType” is “smoothing” and the “standard” is “duration” (time). The “order” field is left blank, or with “NA”.

The most complicated addition to the model in version 2 was the three protocols tables (“protocols”, “protocolRelationships”, and “protocolSteps”). While initially complex, these tables can be adapted to be as simple as users require. Laboratory and sampling protocols are, however, complicated, and representing complex processes recorded in natural language as linked rows of data required some creative thinking. The inclusion of methods information is critical to understanding the data and provides invaluable context for interpreting and aggregating measurements. Moving beyond laboratory protocols, version 3 adds the optional “calculations” table to the model. This addition makes transparent what transformations and calculations have already been applied to the data, and what mathematical and analytical methods were applied to a measure or aggregation. Because the PHES-ODM allows users to record both raw measurements, as well as aggregations and estimates, being able to robustly and transparently report how these calculations were performed is crucial. An entity relationship diagram of the “calculations” table can be found in Figure 10a.

Within the calculations table, the “calculationID” field is the PK for the table. It is generated in practice as a composite key, made by concatenating the “pipelineID” and “treatmentID” fields. The “pipelineID” is the identifier for a data pipeline, or a series of calculations/data treatments. “pipelineID” is also the field that will link to the “measures” table and be referenced there. This ID is the shorthand for the data pipeline used. “treatmentID” is identifier for each data treatment or calculation within the “calculations” table. A series of treatments make up a pipeline, or a pipeline can be a single treatment. The fields “name” and “summary” are optional free text fields to record a name or to summarize data treatment. If used, the “summary” field should explain terms used in the “equation” field. The “calcType” field is a categorical variable used to explain the nature of a data treatment. The valid enumeration values are “normalization”, “standardization”, “smoothing”, or “predictiveModels”. For the “standard” field, users categorically record the standard to which something is standardized (i.e., Pepper Mild Mottle Virus (PMMoV), wastewater flowrate, etc.) or smoothed (i.e., Bayesian smoothing, 7-days, time, etc.). The field is populated by measures and categories available in the larger model. When structuring data treatments in a pipeline, the “order” field uses an integer to structure the flow of treatments within the pipeline. The “equation” field optionally specifies the equation used in the data treatment. The reference link (“refLink”) field provides a link to details on the data treatment. Ideally, this reference should point to a DOI or other persistent digital archiving, such as Zenodo. “sourceCode” records the source code for the data treatment. This field is more applicable for algorithms and complex modelling. Users can record the full code as text or enter a URL to where the code is stored (a different URL than the “refLink” field). Finally, the optional “lastEdited” field and “notes” fields record when data were last updated, and other details.

The “calculations” table works in concert with the new “dataTreat” field in the “measures” table. This new field takes enumeration values and is used as a quick flag to describe the nature of a measure. This helps avoid issues of analysts accidentally using predicted or estimated measures as raw or aggregated data.

As an example, a user recording an estimated measure generated by a predictive algorithm using WWS data standardized to PMMoV in the “measures” table would record the measure normally. The “dataTreat” field in the “measures” table for that measure would take the value “predicted”. In the “calculations” table, the standardization of the WWS data to PMMoV would be recorded as one data treatment, with the “calcType” field recording “standardization” and the “standard” field recording “pmmov”. The “order” field in this row would record “1”, as it is the first step in the pipeline. The predictive algorithm would be recorded as a second treatment, with the “calcType” of “predictiveModels”, and the “standard” left blank or “NA”. The “order” in this row would be “2” as it is the second step in the pipeline. Both rows would share a “pipelineID”. An example of this data entry can be found in Figure 10b. Another example, this time of a single-treatment pipeline, is found in Figure 10c, where a 7-day moving average calculation is done to smooth the WWS data. In the “measures” table, the associated measure would record “derived” in the “dataTreat” field. In the “calculations” table, the “calcType” is “smoothing” and the “standard” is “duration” (time). The “order” field is left blank, or with “NA”.

#### 3.4.6. Implementing Structural Solutions: Site Level and Recording Spatial Resolution

**Figure 11:**
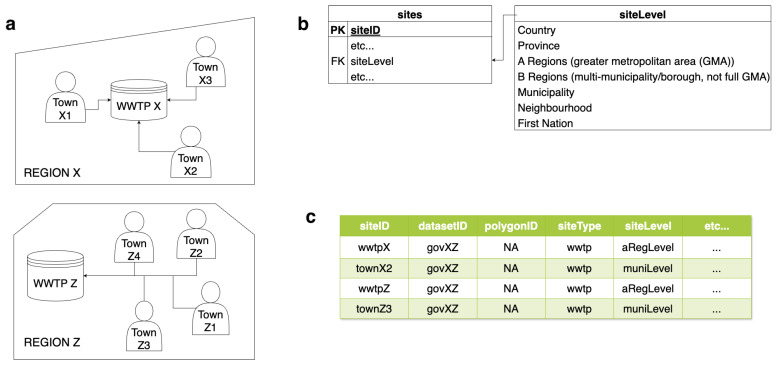
(**a**) An illustration of the regional wastewater infrastructure and flow. The figure shows two regions: Region X where the three towns in the region all pump wastewater to the central wastewater treatment plant (WWTP X), but the infrastructure allows for sampling for each individual town in the region. The other region, Region Z, has all the towns pump their wastewater to WWTP Z, but it is transported in such a way that it is impossible to sample the towns’ contributions separately. (**b**) Valid categories for the new “siteLevel” field. This figure shows the valid categories and where they go in the “sites” table’s new field. (**c**) Example data entry in the “sites” table for Regions X and Z. This table shows how the “sites” table with the new “siteLevel” field would be used for Region X and Z.

The issue of unreported differences in the spatiotemporal resolution of a measure, particularly in aggregated data sets, is an important one. The new “calculations” table helps to some extent by providing details on standardization or aggregation calculations. It is still, however, important to know what geography a sample or measure is intended to reflect. To address this, the PHES-ODM has always stored polygon information, capturing the exact geography or catchment boundaries for a site. In situations where polygon information is not available, or where it is unclear whether the area represented is a city or a region, problems persist. For example, let us say that wastewater treatment plant X (WWTP X) is a WWTP that services REGION X: a region in which there are three municipalities. There is also WWTP Z, which is the wastewater treatment plant that services REGION Z: a region in which there are four municipalities. With the way that the wastewater infrastructure is built, it is possible to sample WWTP X such that you can have measures that reflect each of the municipalities individually, or the whole region. Conversely, WWTP Z can only be sampled and measured in a way that reflects the whole region, not individual municipalities (Figure 11a). When it comes to analysis and aggregation, it is useful to users to be able to differentiate between the geographic level that is being sampled (whether that is a region, or something smaller) so that comparisons can be more meaningful. For example, comparing the measures from Town X2 and Region Z as though they are the same type of geography would not be appropriate. While comparison at this level is allowed, making explicit that there is a spatial resolution difference is essential. This means ensuring future data users know that it is not possible to get any granular data from Region Z at the municipal level.

To address this, “siteLevel” is a new field added to the “sites” table to eliminate this kind of ambiguity. The valid categorical values for “siteLevel” are shown in Figure 11b and are: country level aggregation, where the measures reflect an entire country; province level aggregation, where the measures reflect an entire province or state; A regions, where the measures reflect an entire greater metropolitan area (GMA) made up of several smaller municipalities; B regions, where the measures reflect multiple municipalities that are a part of a shared GMA, but do not reflect a GMA in its entirety; municipality level, where the measures reflect a single municipality; neighbourhood level, where the measures reflect a single neighbourhood; and First Nations level, where the measures reflect a single First Nation. An example data entry for the example in Figure 11a can be found in Figure 11c.

#### 3.4.7. Implementing Structural Solutions: Robustness vs. Ease of Use

One final issue with data models is balancing ease of use with robustness. This is a challenge for all data standards, and one that we have tried to balance since the beginning. The first version of the PHES-ODM was very straightforward and easy to use but was very limited in what it could report. Version 1 could still work today for very basic reporting of SARS-CoV-2 detection. As the field of WWS matured, however, additional targets needed to be added, along with different measures of the different targets, additional metadata, and more context. With version 2 and now version 3 of the model, we have striven to be as robust a model as possible and included everything that was asked of us. With this, however, the complexity has grown. Today, when interacting with new users, it is not uncommon to hear that the model is somewhat intimidating, and they worry about the time needed to understand and adopt the model. To respond to this issue, however, we have made a robust documentation website [32] and video tutorials [31] available so that users can feel more comfortable with the model right away. We also have a message board hosted on Discourse [30] where users can ask questions, and communications about new developments to the model are openly discussed. New issues for new parts can be submitted on GitHub [71], and we are starting to explore AI-supported tools to empower users to get started with as low of a barrier as possible. However, we understand that these support tools and resources do not automatically make up for the complexity of the model. To this end, we hope to conduct future work to assess the ease of use more empirically, via a usability study.

The PHES-ODM has structural provisions to address issues around data definitions, and around balancing robustness against ease of use. These are addressed through the larger support structure around the model, rather than the structure of the model itself. This larger support, documentation, and education ecosystem around the PHES-ODM also helps to address issues around understanding the data and its primary use, and around understanding the classification and coding systems of the values used in the model structure.

#### 3.4.8. Implementing Structural Solutions: Expanding Data Relationships

As a relational database structure, the PHES-ODM works to represent real-world relationships in the data. In version 2 of the data model, in addition to the relational linkages between tables, additional tables were added to record more complex relationships, and many “parent” fields were added. Therrien et al. cover the structure of the relationships tables very well [14]. The parent fields in version 2 (namely Parent Dataset ID, Parent Site ID, and Step Provenance ID) served a similar purpose to the relationships tables, but on a much simpler level. They allowed sites or datasets to be contained within larger sites or datasets, making geography and data ownership levels more explicit. The Step Prevenance ID also helped to make a type of citation possible by linking related or adapted protocol steps.

**Figure 12:**
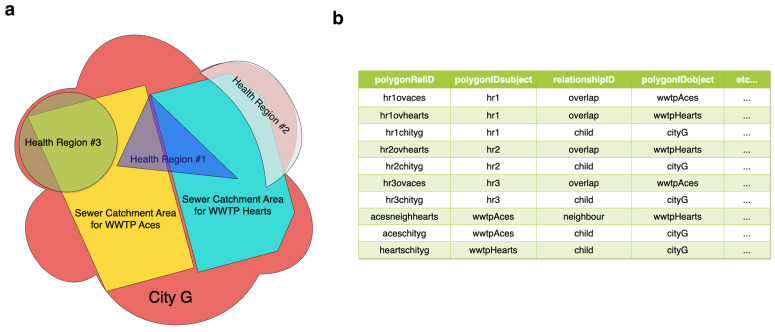
(**a**) An illustration of fictional polygons within a city. The figure shows a city (City G) that is served by two shown sewer catchment areas (area Aces and area Hearts). There are also three health regions shown in the city, each with partial overlap with at least one of the sewer catchment areas. (**b**) polygonRelationships table input for the polygons in City G. This figure shows the polygonRelationships table populated to describe the relationships between all the polygons in City G.

In version 3 we have added one new relationships table, and two new parent-type fields. The new relationship table is “polygonRelationships”, which allows for overlapping polygons, or nested polygons, to have that relationship made explicit. The impetus for the addition of this table was that different agencies may use different polygons to cover different areas. For example, a health region may partially overlap with a wastewater catchment area, or it may be entirely contained within it. There may even be multiple health regions within a sewage catchment area, but they are both fully contained within a city. An example of overlapping polygons is found in Figure 12a. Making the overlapping spatial geometry more explicit was something users said was essential for their data management, and so the polygon relationship table was born. An example of the polygonRelationships table being populated for the example of City G can be found in Figure 12b. The polygonRelationships table can be read in the formula: “[polygonIDsubject] is [relationshipID] to [polygonIDobject]”. For example, from row one of Figure 12b, Health region #1 (hr1) is overlapping WWTP Aces’ catchment area (wwtpAces). The “polygonRelID” is the unique identifier for each row, and the PK for that table.

Within two of the new tables added in version 3 there are two fields operating like parent fields to group rows together from within a table. These two fields are the “actionGrpID” from the public health actions table, and the “pipelineID” from the calculations table. We think a balanced approach to intra-table grouping for defining relationships, and external table relationship mapping for more complex cases allows users to record complex linkages as simply as possible, preserving detailed metadata without overburdening data generators.

#### 3.4.9. Implementing Structural Solutions: Future Directions

While we believe that the PHES-ODM fills a very important niche in the WWS data ecosystem, the work of our mission to improve WWS and epidemiology, through interoperable, transparent, and efficient data collection and use, remains a work in progress. As new programmes emerge and as the field continues to grow and evolve, the data architecture required to support these programmes must also adapt. Part of this evolution for the PHES-ODM involves continuing to strengthen and develop a decentralized governance model and further research into assessment of the model; particularly examining ease of use, and semantic data loss during mapping processes.

Other work to be done in the future is the development of additional tools, and structural improvements to the model. Tools to better automate the transition from narrative protocol information to PHES-ODM formatted protocols would be an important addition. As far as the structure of the model is concerned, as mentioned above, developing stronger guardrails to ensure data can be effectively embargoed within the model is one area for further development. Other areas for model expansion include developing stronger data storage for WWS sequencing data, better integration of continuous and timeseries data, and stronger provisions for data versioning.

Currently, sequencing metadata can be stored in the PHES-ODM, and aggregate-level details of variant or mutation presence and proportion can be recorded, along with site and sample details. The raw sequencing data must be stored elsewhere, however, and referenced externally through the accessions table. Similarly, raw time series data is difficult to integrate into the PHES-ODM, even in version 3.

Continuous sensor measurement data can be integrated into the PHES-ODM by recording measures without a sample identifier. For example, a flow meter in a wastewater treatment plant takes a flow measure at a given time, but it is not linked to a discrete sample. Matching of date windows and site identifiers allows the linkage of other measures with this information. Similarly, some current users of the model store more aggregated data in the model, because almost all expected samples are composite in nature. This allows them to store the aggregate associated with the sample, but not the raw time-series values to reduce unnecessary data bulk. Improving this structure moving forward will be a priority.

Revisiting the protocols tables and the calculations table is also something we are looking to do. The protocols tables are very complex for many users, and with calculations, we intended to keep the structure simple, which can also present issues of imprecision and user input error. Much like how we are using LinkML [29] to provide an alternate description or source file for the structure of the PHES-ODM, we may choose to employ the Common Workflow Language (CWL) [72] or Workflow Description Language (WDL) [73] to better standardize some of these tables. This is an item, however, that treads the line of continuing to try to keep the model accessible to all (using Excel/CSV files, using natural language, providing robust documentation) with responding to issues with sophisticated solutions (requiring users to be familiar or become familiar with additional workflows and markup languages). Challenges in this regard are always present, and that is another reason why the work of developing the PHES-ODM remains on-going.

## 4. Conclusions

WWS, as it scales up globally, is poised to save many lives as a valuable tool in the global public health surveillance toolkit. The utility of surveillance is, however, in the data it generates, and that data is only valuable insofar as it is useful and useable. The FAIR data principles, among other data justice principles like data feminism, cognitive interoperability, and the EOSC interoperability framework, provide a useful metric to which WWS data can aspire toward. In meeting these principles, WWS data can uphold data justice and do the greatest good for the largest number of people. While there are many paths to be taken to accomplish and adhere to these principles, using the PHES-ODM as a database structure is a well-supported way to accomplish these goals.

Beyond just supporting use and re-use, as well as interoperability and accessibility, the model also exists with various support tools to makes its adoption and use as easy as possible for users. This includes robust written [32] and video documentation [31], a message board [30], as well as sharing [70], validation [40,69], and mapping tools [62]. This means that if a user starts with the model today, they have the information at their disposal to get started within minutes and have the infrastructure on hand to validate and share their data, without any investment of their own development time. The model is entirely modular and scalable as well, so users can start with a very basic programme and data template, and the model will expand and grow with their programme as it develops.

As we continue in a global age of polycrisis [25], there will be increasing political pressure and competition regarding which issues to prioritize. The COVID-19 pandemic was one arm of the polycrisis, and it fueled a great deal of advancement in public health preparedness. Not least of these developments was the establishment of many WWS programmes globally, and the convening of many expert research groups. Even major global non-profit agencies, like the Bill & Melinda Gates and the Rockefeller Foundations have taken up the banner of WWS establishment in low-resource settings [7,8]. As the needle shifts, however, and different aspects of the polycrisis pull more focus, we find ourselves at the nadir of WWS. As many smaller programmes close (as almost all provincial WWS programmes in Canada have closed, transferring responsibility to the federal programme), funding priorities shift away from health research, and public interest shifts away from surveillance, the hard-won progress in WWS faces an existential threat.

Ensuring that the data generated by these programmes remain useful and of high quality is now essential for cementing WWS programmes for the future. All surveillance programmes already have data reporting standards and dictionaries. There is often, as is the case with LOINC and SNOMED, widespread global adoption of these dictionaries in other health surveillance systems. This not only allows for care integration within hospitals and health regions, but also across regions and even globally. “Futureproofing” WWS and its data is already a focus in several major coalitions and action groups; there is a GLOWACON technical working group [9], an ELIXIR working group [74], and the WHO is working on data-related projects and questions [75], among many others. What is often the focus of these groups, however, are analytical and quality suggestions, modelling approaches, and what data to record. The last item is one relevant to the work of the PHES-ODM, but there is a noted lack of advice and focus on how to record data. The field has changed since the PHES-ODM was first developed and there are now several data models and standards that act as strong candidates for standardizing WWS data. With the PHES-ODM being used in Canada and the EU [17,27], NWSS being used in the United States, and other models being used in other programmes, there is adoption of some standards. The problem has instead shifted from one of a lack of options, to one of a lack of adoption. Even with the launch of the much-expanded version 2 of the PHES-ODM, there were already other models and dictionaries being prepared, with different strengths and focuses. While the PHES-ODM has focused more on PCR testing, the PHA4GE format is very focused on sequencing data, for example (a larger comparison summary can be found in Table 2). There is a gap between the perceived need and the action on WWS data, and our hope is that by continuing to share the PHES-ODM, its strengths, and its ease of use, that we can help make WWS data standardization and interoperability a reality.

Barriers to model and standard adoption seem to be based in a lack of interest in adopting an external standard. Other challenges are that these labs and programmes are often busy and lack the time and resources to devote to learning and switching over to a different data model. For new programmes, perhaps information on the importance of standard adoption and what options are available are not as readily accessible. The PHES-ODM strives to counter some of these with robust documentation, video walkthroughs, premade templates, and the publication and distribution of model information. The discussion boards, emails, and participation in global networks also works to support adoption, though it will always require interest and investment from other parties. With standards such as LOINC, institutional or regulatory mandates have pushed greater adoption. With the PHES-ODM and other WWS standards, this has happened in some settings—for example, for the Ontario programme, labs were required to use version 1.1 of the model. Besides regulatory mandates, developing other adoption incentives is still important, and that is one of the main reasons why the PHES-ODM team develops many support libraries and tools. Having the PHES-ODM exist as an ecosystem with pre-developed tools to automate and ease data-related tasks is one way in which we hope to incentivize adoption.

The issues facing WWS today, particularly in the realm of data, require structural solutions. Relying on the prescience of over-burdened data generators, or their goodwill and ability to go above and beyond will not lead to success. These programmes and individuals are already overburdened, but by providing instruction, support, and tools, we can solidify these programmes, their data, and the advances made in the last 6 years for the benefit of future generations.

## Figures and Tables

**Table 1:** Recommended variables in the core PHES-ODM tables. Identifiers (in italics) are mandatory and uniquely identify a row in their home table; the others are strongly recommended for most programmes.

Table	Variable	Description
Sites	*Site ID*	Unique identifier for the location where a sample was taken.
	Site name	Human-readable name of the site.
	Sample shed	Geographic area, physical space, or structure from which a sample is taken.
	Site type	Type of site or institution where the sample was taken.
Samples	*Sample ID*	Unique identifier for a sample.
	*Site ID*	Site where the sample was collected.
	Sample material	Type of sample.
	Sample collection type	The type of collection (e.g., grab, composite).
	Collection date time	Date, time, and time zone the sample was taken (or start of the collection period).
Measures	*Measure Report ID*	Unique identifier for a measurement.
	*Sample ID*	Sample to which the measure refers (optional for site-level measures such as flow).
	Analysis date start	Date the measurement or analysis was started.
	Measure	A measurement or observation of any biological, physical, or chemical substance.
	Value	Value of a measure, observation, or attribute.
	Unit	Units of measurement

## Data Availability

All data and documentation presented in this paper is openly available. Details, tables, and resources can be found at the project website (https://www.phes-odm.org, accessed on 3 March 2026), the GitHub repository for the project (https://github.com/PHES-ODM/PHES-ODM, accessed on 3 March 2026), or the Open Science Foundation repository (https://osf.io/ab9se/overview, accessed on 3 March 2026). Any further queries for support can be directed to the Discourse message board (https://odm.discourse.group, accessed on 3 March 2026), or the corresponding author.

## Abbreviations

The following abbreviations are used in this manuscript:

CDM	Common Data Model
COD	Chemical Oxygen Demand
CWL	Common Workflow Language
EDGE	Enterics, Diagnostics, Genomics & Epidemiology
FAIR	Findable, Accessible, Interoperable, and Reuseable
FHIR	Fast Health Interoperability Resources
FK	Foreign Key
GIS	Geographic Information System(s)
GISAID	Global Initiative on Sharing All Influenza Data
GMA	Greater Metropolitan Area
HL7	Health Level Seven International
ICHI	International Classification of Health Interventions
LOINC	Logical Observation Identifiers Names and Codes
MIxS	Minimum Information about any (x) Sequence
NH_4_^+^-N	Nitrogen present as ammonium
NWSS	National Wastewater Surveillance System
OHDSI	Observational Health Data Sciences and Informatics
OMOP	Observational Medical Outcomes Partnership
PCR	Polymerase Chain Reaction
PHA4GE	Public Health Alliance for (4) Genomic Surveillance
PHAC	Public Health Agency of Canada
PHES-EF	Public Health and Environmental Surveillance Evaluation Framework
PHES-ODM	Public Health and Environmental Surveillance Open Data Model
PK	Primary Key
PMMoV	Pepper Mild Mottle Virus
RKI	Robert Koch Institute
SSSOM	Simple Standard for Sharing Ontological Mappings
TSS	Total Suspended Solids
UBA	Umweltbundesamt (German Federal Environment Agency)
USCDC	United States Centers for Disease Control and Prevention
WDL	Workflow Description Language
WWE	Wastewater Epidemiology
WHO	World Health Organization
WHO-FIC	World Health Organization Family of International Classifications
WWS	Wastewater Surveillance
WWTP	Wastewater Treatment Plant
